# Blocking TBK1 alleviated radiation-induced pulmonary fibrosis and epithelial-mesenchymal transition through Akt-Erk inactivation

**DOI:** 10.1038/s12276-019-0240-4

**Published:** 2019-04-15

**Authors:** Hongjin Qu, Lei Liu, Zhe Liu, Hongran Qin, Zebin Liao, Penglin Xia, Yanyong Yang, Bailong Li, Fu Gao, Jianming Cai

**Affiliations:** Department of Radiation Medicine, Faculty of Naval Medicine, Second Military Medical University, 800, Xiangyin Road, 200433 Shanghai, P. R. China

**Keywords:** Respiratory tract diseases, Experimental models of disease

## Abstract

As a common serious complication of thoracic radiotherapy, radiation-induced pulmonary fibrosis (RIPF) severely limits radiation therapy approaches. Epithelial–mesenchymal transition (EMT) is a direct contributor to the fibroblast pool during fibrogenesis, and prevention of EMT is considered an effective strategy to inhibit tissue fibrosis. Our previous study revealed that TANK-binding kinase 1 (TBK1) regulates EMT in lung cancer cells. In the present study, we aimed to investigate the therapeutic potential of targeting TBK1 to prevent RIPF and EMT progression. We found radiation-induced EMT and pulmonary fibrosis in normal alveolar epithelial cells and lung tissues. TBK1 knockdown or inhibition significantly reversed EMT in vivo and in vitro and attenuated pulmonary fibrosis and collagen deposition. Moreover, we observed that TBK1 was elevated in a time- and dose-dependent manner by radiation. Meanwhile, radiation also induced time- and dose-dependent activation of AKT and ERK, each of whose inhibitors suppressed radiation-induced EMT. Intriguingly, silencing of TBK1 with shRNA also blocked the radiation-induced activation of AKT and ERK signaling. The ERK inhibitor did not obviously affect the expression of TBK1 or phosphorylated AKT, while AKT inhibition suppressed activation of ERK without changing the expression of TBK1. Finally, we found that a TBK1 inhibitor inhibited inflammatory cytokine expression in a RIPF model and Amlexanox protected normal cells and mice from ionizing radiation. In conclusion, our results indicate that the TBK1–AKT–ERK signaling pathway regulates radiation-induced EMT in normal alveolar epithelial cells, suggesting that TBK1 is a potential target for pulmonary fibrosis prevention during cancer radiotherapy.

## Lung cancer: Suppressing fibrosis during radiotherapy

The risk of scarred tissues and respiratory distress during radiation treatment of lung cancer could be reduced by targeting an enzyme that alters healthy cells. Lung cancer radiotherapy often causes pulmonary fibrosis, excessive growth of fibrous tissues in the lungs, involving the transition of normal epithelial cells into an invasive form of multipotent stem cells. The development of pulmonary fibrosis limits the clinical application of radiotherapy. Hongjin Qu and co-workers at the Second Military University in Shanghai, China, previously demonstrated that the TANK-binding kinase 1 (TBK1) enzyme regulates this transition. Now, the team have shown that levels of TBK1 itself increased during radiation treatment, together with two proteins that would normally suppress alterations in healthy cells. Inhibiting TBK1, both in cell cultures and mouse models, reversed the cell transitions and prevented pulmonary fibrosis.

## Introduction

Lung cancer is the most common malignant cancer with high incidence and (especially non-small cell lung cancer (NSCLC)) has become the leading cause of cancer-related death^1,2^. Radiotherapy, an effective treatment modality for thorax-associated neoplasms, is recommended as a mainstay in the treatment of NSCLC^3,4^. However, the risk of radiation-induced lung injury (RILI) in normal tissues hampers the efficacy of lung cancer radiotherapy. Accordingly, radiation-induced pulmonary fibrosis (RIPF), the adverse late effect of RILI, limits further application of radiotherapy with increasing radiation doses^3–6^. However, there is no available treatment strategies against RILI, and the underlying mechanism remains unclear^7,8^.

Epithelial–mesenchymal transition (EMT) plays a critical role in pathological fibrosis in many tissues. It was also reported that EMT occurs in idiopathic and experimental lung fibrosis^9,10^. Activated fibroblasts originating from alveolar epithelial cells through EMT produce collagen and extracellular matrix proteins in the lung interstitium, which results in lung fibrosis^11–14^. Radiation-induced EMT may play an important role in RIPF. Furthermore, overexpression of EMT-associated genes or promotion of radiation-induced EMT accelerates the incidence of RIPF^15,16^.

EMT is characterized by downregulation of epithelial proteins, such as E-cadherin, and acquisition of mesenchymal markers, including vimentin and α-smooth muscle actin (α-SMA)^17^. The regulation of classical EMT centers on the activities of major transcription factors, such as SNAI1, SNAI2, ZEB1, ZEB2, and TWIST1, which have been described and reviewed extensively^18,19^. TANK-binding kinase 1 (TBK1) has been identified as a downstream effector of miR-200c, which inhibits EMT by directly targeting ZEB1 and ZEB2, and our previous findings revealed that TBK1 signaling regulates radiation-induced EMT by controlling GSK3β phosphorylation and ZEB1 expression in lung cancer cells^14,20,21^. However, the exact role of TBK1 in radiation-induced EMT, especially in normal alveolar epithelial cells and tissues, still needs further exploration.

In recent studies, the AKT and ERK signaling pathways have been demonstrated to be the cardinal signaling programs mediating EMT in cancer cells^22–25^. It is well known that TBK1 can activate AKT through direct phosphorylation^26,27^. Furthermore, it was revealed that radiation-induced EMT in alveolar type II epithelial cells is mediated by the ERK/GSK3β/Snail pathway^13^. We hypothesized that TBK1 might regulate radiation-induced EMT through the AKT signaling pathway or ERK signaling pathway. In this study, we explored the possible mechanisms associated with TBK1 in radiation-induced EMT in normal alveolar epithelial cells. Our data indicate that TBK1 may act as an upstream trigger of AKT and ERK via phosphorylation to affect the radiation-induced EMT process in normal alveolar epithelial cells, suggesting that TBK1 is a potential target for treatment of RIPF.

## Materials and methods

### Cell culture and treatment

Cells of the rat alveolar type II epithelial cell line RLE-6TN were purchased from the American Type Culture Collection (Manassas, VA, USA) and routinely maintained in Dulbecco’s modified Eagle’s medium (DMEM)/F12 (Gibco, Grand Island, NY, USA) with 10% fetal bovine serum (Gibco), 100 IU/ml penicillin, and 100 μg/ml streptomycin (Invitrogen, CA, USA) at 37 °C with 5% CO_2_. For some conditions, cells were pretreated with inhibitors for 2 h before irradiation.

### Mice and treatments

Female C57BL/6 mice, 7 weeks of age, were purchased from the Experimental Animal Center of Chinese Academy of Sciences (Shanghai, China). Mice were randomly divided into four groups as follows: group 1, nonirradiated control (Control); group 2, irradiation + dimethyl sulfoxide (DMSO) (infrared (IR)); group 3, irradiation + amlexanox (IR + Amlexanox); group 4, irradiation + prednisone (IR + Prednisone). All mice were housed under standard laboratory conditions and were allowed to acclimate to the animal center for 1 week prior to treatment. Amlexanox was obtained from Abcam (Cambridge, MA, USA) and given orally 3 days before irradiation at a dose of 50 mg/kg/day and maintained until 4 weeks after irradiation. Prednisone (SHANGHAIZZBIOCO, Ltd., Shanghai, China) was administered orally at 5 mg/kg/day postirradiation for 4 weeks.

### Irradiation

Cells and mice were irradiated with γ-rays using a ^60^Co irradiator (Faculty of Naval Medicine, Second Military Medical University, China). Cells were irradiated with a single dose of 0, 2, 4, 6, 8, or 10 Gy at a dose rate of 1.8 Gy/min. After anesthetization with 10% choral hydrate (350 mg/kg), the mice were put in a holder designed to immobilize anesthetized mice so that only the whole thorax was exposed to the beam. Mice received a single dose of 20 Gy (thoracic irradiation) at a dose rate of 1 Gy/min. All irradiations were performed at room temperature.

### Western blot analysis

We used Proteo-JETTM Mammalian Cell Lysis Reagent (Thermo) to lyse cells. Crude lysates were centrifuged at 14,000*g* for 20 min at 4 °C. Lysate proteins were mixed with loading buffer and denatured by heating at 100 °C for 10 min. Proteins were resolved by SDS-PAGE and transferred to a PVDF membrane (Millipore, Billerica, MA, USA), blocked with 5% nonfat milk, and then incubated with primary antibodies overnight at 4 °C on a shaking table. Primary antibodies targeting E-cadherin (1:1000), Vimentin (1:1000), AKT (1:1000), AKT-pS473 (1:1000), AKT-pT308 (1:1000), ERK (1:1000), p-ERK (1:1000) (Cell Signaling Technology, Danvers, MA, USA), α-SMA (1:1000), TBK1 (1:1000) (Abcam, Cambridge, MA, USA), and GAPDH (Beyotime, China) were used. Then, the blots were incubated with the secondary antibody, HRP-conjugated IgG (Beyotime, dilution 1:5000), for 1 h at room temperature. Specific bands for each protein were detected with an Image Quant LAS4000 imager (GE Healthcare Life Science, Pittsburgh, PA) using Amersham ECL (Millipore). Densitometric analysis of expression was performed using ImageJ software (National Institutes of Health, Bethesda, MD). The expression of each molecule was normalized to GAPDH expression.

### Hematoxylin-eosin and Masson’s trichrome staining

The right lung of the mice was harvested and fixed in 4% neutral paraformaldehyde and then embedded in paraffin. Tissue specimens were sectioned at a thickness of 5 mm and conventionally stained with hematoxylin-eosin (H&E) and Masson’s trichrome stain for histological examination. Fibrosis grade was evaluated blindly using a semiquantitative scoring system.

### Hydroxyproline assay

The concentration of hydroxyproline was measured with a hydroxyproline assay kit (Nanjing Jiancheng Bioengineering Institute, Nanjing, China) according to the manufacturer’s protocol. Hydroxyproline content was calculated according to total lung weight and is expressed as micrograms in the lung.

### Clonogenic assay

The radiation protective effect of amlexanox was assessed with clonogenic assays. The cells were trypsinized, counted, and seeded in 60-mm culture dishes in two sets of three for each dose of radiation. Amlexanox, at a final concentration of 25 µM, was added to the cell dishes 1 h before irradiation and was discarded 4 days after irradiation. Sufficient numbers were seeded to ensure that approximately 30–100 macroscopic colonies would appear in each plate after 10–14 days. Colonies were stained with 0.5% gentian violet in methanol and counted. The plating efficiency (PE) for each dose was calculated by dividing the number of colonies by the number of cells plated and expressing the result as a percentage. The surviving fraction was calculated by dividing the PE of the treatment by the PE of the appropriate unirradiated control.

### Cell proliferation assay

Cell proliferation was determined using Cell Counting Kit-8 (Dojindo, Kumamoto, Japan). Cells were suspended and seeded into 96-well plates at 5 × 10^3^ cells/well. Then, 48 h after irradiation, cell proliferation was assessed with a CCK-8 assay.

### Apoptosis analysis

Apoptosis of cells treated with or without amlexanox and irradiation was determined by Annexin V-FITC (AV) and PI staining. The cells were plated in six-well plates at a density of 10^5^ cells per well and allowed to attach for 24 h. The cells were pretreated with and without 25 µM amlexanox before irradiation. After 24 h, the cells were harvested by trypsin digestion, washed with precooled phosphate-buffered saline (PBS) twice, and resuspended. The cells were stained with AV and PI at room temperature for 15 min in a dark room according to the Annexin V-FITC Apoptosis Detection Kit (BD Pharmingen, San Diego, CA) instructions.

### Immunofluorescence staining

Cells cultured in six-well plates were irradiated with a single dose of 8 Gy of ^60^Co γ-rays. After 1 h or 48 h, cells were washed with PBS and fixed in 4% paraformaldehyde for 20 min. Then, we washed cells with PBS and permeabilized the plasma membrane of cells with 0.1% Triton X-100 for 30 min at room temperature. Cells were blocked with 5% bovine serum albumin (BSA) for 1 h and incubated with E-cadherin, Vimentin (Cell Signaling Technology), α-SMA, TBK1, or p-AKT (Abcam) antibodies at 4 °C overnight. After being washed with PBS, the sections were incubated with Alexa Fluor 488-conjugated anti-mouse (Invitrogen) and Texas red-conjugated anti-rabbit (Vector Laboratories, Burlingame, CA, USA) antibodies at room temperature for 30 min. Nuclei were counterstained with 4′,6-diamidino-2-phenylindole (DAPI), and the slides were analyzed using a fluorescence microscope (Nikon, Tokyo, Japan). For the lung tissue, after deparaffinization and antigen retrieval, antibodies against E-cadherin and α-SMA were mixed and used for immunofluorescence staining.

### Enzyme-linked immunosorbent assay

Bronchi alveolar lavage fluid (BALF) was collected, and interleukin-4 (IL-4) and interferon-γ (IFN-γ) levels were determined using a commercially available ELISA (enzyme-linked immunosorbent assay) kit according to the manufacturer’s instructions. The OD value was determined at 450 nm using a Varioskan LUX Multimode Microplate Reader (Thermo Fisher Scientific Inc. Waltham, MA, USA) and calculated at the linear portion of the curve. BALF cytokine levels were measured using a commercial ELISA kit from Anogen (Mississauga, Ontario, Canada).

### Mouse survival analysis

To determine the radioprotective property of amlexanox, mice were divided into the following two experimental groups: group 1, radiated (whole body) only animals (*n* = 10, 20), animals were treated with vehicle (DMSO, 0.2 mL, p.o.) 1 h before irradiation; group 2, 50 mg/kg amlexanox + irradiated animals (*n* = 10, 20), animals were treated with 50 mg/kg amlexanox (0.2 mL, p.o.) 1 h before irradiation. After exposure, the mice were maintained at the animal facility, and mortality and morbidity was observed for 30 days.

### Lentiviral short hairpin RNA (shRNA) production/infection

Rat TBK1 shRNA (CTGGGTGAGATTTCAGACATA) and scrambled shRNA (TTCTCCGAACGTGTCACGT) lentiviruses (made from the vector GV248) were generated by BioLink (Shanghai, China). TBK1 shRNA lentiviral vectors were used for knockdown of TBK1. Cells were seeded at 1 × 10^5^ cells/well into six-well plates and infected with lentiviral particles using polybrene (10 mg/mL). After infection, virus-containing medium was replaced with normal medium, and then cells were selected with puromycin (2 mg/mL).

### Statistical analysis

Statistical analyses were performed using Prism 5.0 software (GraphPad). All quantitative data are presented as the mean ± SEM and were obtained from at least three independent experiments. For normalization, sham control values were set to 100%, and values for irradiated animals are displayed as a percent of the sham control values. Student’s two-tailed unpaired *t-*tests were used to compare differences between two groups. One-way analysis of variance (ANOVA) followed by Newman–Keuls multiple comparison tests were used to compare more than two groups. Two-way ANOVA with post hoc Bonferroni multiple comparison tests were used to compare groups split on two independent variables. Statistical significance was set at *P* < 0.05.

## Results

### TBK1 upregulation participates in radiation-induced EMT

**Fig. 1:**
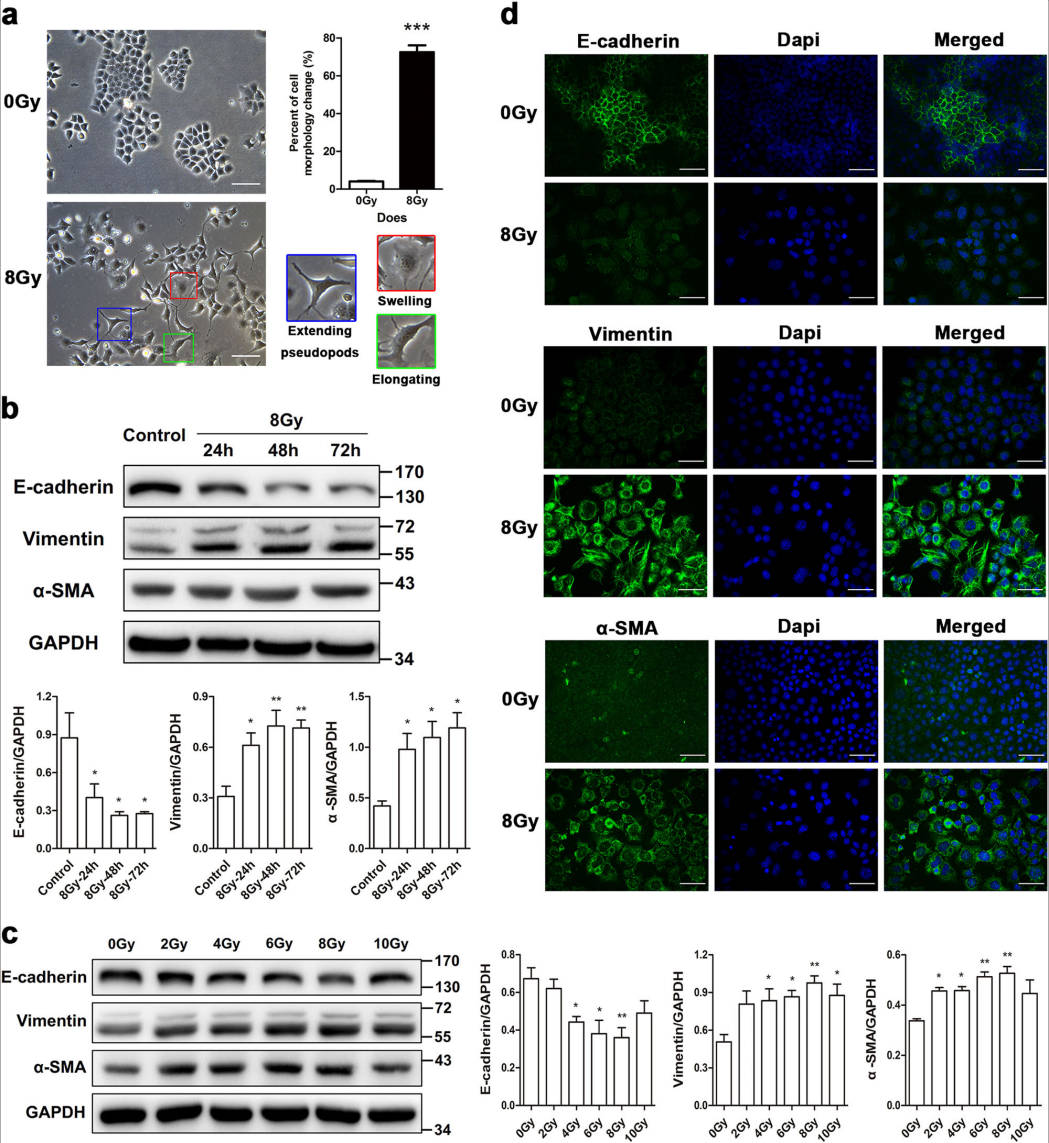
Radiation induces changes in cell morphology and EMT-associated protein expression in RLE-6TN cells. Cells were irradiated with a single dose of 2, 4, 6, 8, or 10 Gy ^60^Co γ-rays, and cell morphology and EMT-associated protein markers were observed at 24, 48, and 72 h postirradiation. **a** Representative images of cell morphology (photographed at 48 h after 8 Gy irradiation or nonirradiation). Cells suffering morphologic changes were counted in random microscope fields according to whether cells became swollen (red outline), elongated (green outline), or exhibited extended pseudopodia (blue outline) compared with a cuboidal appearance, and the percentage was calculated. Scale bar represents 100 μm. The data are presented as the mean ± SEM (*n* = 10). ***P < 0.0001 vs. nonirradiated control. **b**, **c** Representative western blots and densitometric quantification of E-cadherin, vimentin, and α-SMA protein levels. GAPDH was used as the loading control. The data are presented as the mean ± SEM (*n* = 3). *P < 0.05 and **P < 0.01 vs. nonirradiated control. **d** Immunofluorescence staining for E-cadherin, vimentin, α-SMA (green), and DAPI (blue) in nonirradiated control and irradiated cells at 48 h postirradiation. Scale bar represents 50 μm

Rat type II alveolar RLE-6TN cells were irradiated with a single dose of 0, 2, 4, 6, 8, or 10 Gy γ-rays, and cell morphology was observed at 0, 24, 48, and 72 h postirradiation. We found that 70% of the cells changed from a cuboidal appearance to a swollen and elongated morphology with extended pseudopodia after irradiation, especially at 48 h postirradiation with 8 Gy γ-rays (Fig. 1a). To test if these changes were in line with EMT-associated proteins, we used western blotting to analyze expression of the epithelial marker E-cadherin and mesenchymal markers Vimentin and α-SMA. A decrease in the protein level of E-cadherin and an increase in vimentin and α-SMA were observed in cells after irradiation (Fig. 1b, c). Western blotting data revealed significant variation in the markers at 48 and 72 h postirradiation compared with the nonirradiated group (Fig. 1b). Intriguingly, changes in EMT-associated proteins in cells irradiated with 10 Gy were less marked than in those irradiated with 8 Gy (Fig. 1c). Moreover, the changes in EMT-associated markers were also confirmed by immunofluorescence staining (Fig. 1d). Taken together, these data suggest that irradiation likely induces EMT in RLE-6TN cells, especially at 48 h postirradiation and a γ-ray dose of 8 Gy.

**Fig. 2:**
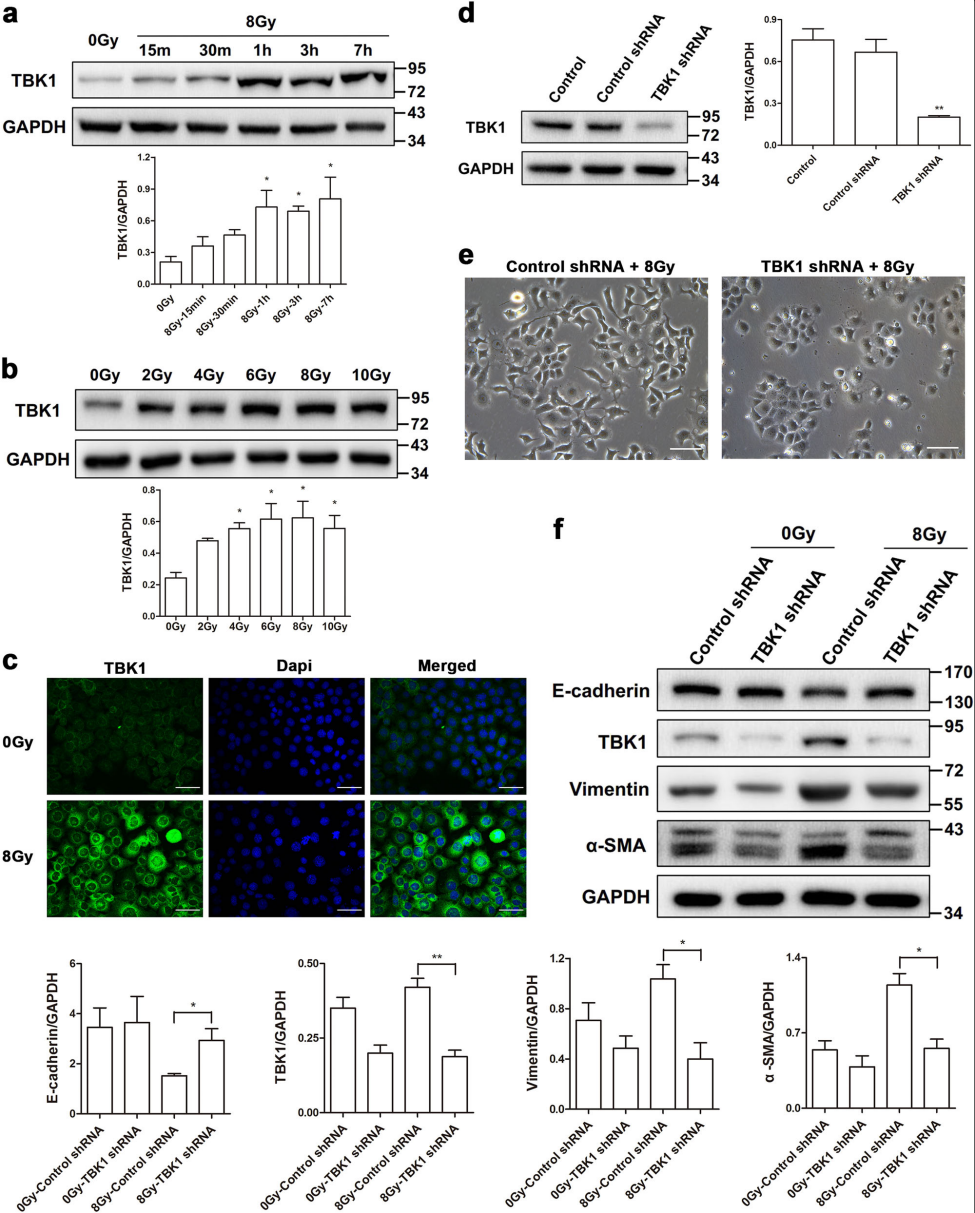
Radiation induced TBK1 expression and knockdown of TBK1 attenuates radiation-induced EMT. **a**, **b** Representative western blots and densitometric quantification of TBK1 protein levels at different times and with different radiation doses. **c** Immunofluorescence staining for TBK1 (green) and DAPI (blue) in nonirradiated control and irradiated cells at 1 h postirradiation. Scale bar represents 50 μm. **d** Representative western blot and densitometric quantification of TBK1 following infection with TBK1-specific shRNA lentivirus to confirm target suppression. **e** Phase contrast microscopy images of cells expressing TBK1-specific shRNA and control shRNA at 48 h after 8 Gy irradiation. Scale bar represents 100 μm. **f** Western blot analysis of E-cadherin, vimentin, and α-SMA expression in TBK1 knockdown RLE-6TN cells. GAPDH was used as the loading control in all the above western blot analyses. All data are presented as the mean ± SEM (*n* = 3). *P < 0.05 and **P < 0.01 vs. nonirradiated control

To investigate the role of TBK1 in EMT in normal type II alveolar epithelial cells, we first determined the radiation responsive property of TBK1. We subjected RLE-6TN cells to a single dose of 8 Gy and found a time-dependent increase in the TBK1 protein level, especially after 1 h (Fig. 2a). Then, we observed a significant dose-dependent increase in TBK1 protein from 2 to 10 Gy (Fig. 2b). To further validate the upregulation of TBK1, we conducted an immunofluorescence assay, which showed more fluorescence after 8 Gy irradiation at 48 h in contrast with the nonirradiated control (Fig. 2c). After that, we generated TBK1-specific shRNA to silence the expression of TBK1, and a significant reduction was observed in TBK1 expression (Fig. 2d). We found that most TBK1 knockdown cells exhibited a changed morphology, from a swollen, elongated morphology with extended pseudopodia to a cuboidal appearance at 48 h postirradiation (Fig. 2e). Moreover, TBK1 knockdown increased the expression of the epithelial marker E-cadherin and decreased the expression of the mesenchymal markers vimentin and α-SMA after irradiation (Fig. 2f). Overall, overexpression of TBK1 contributed to radiation-induced EMT of RLE-6TN cells, and the loss of TBK1 expression may reverse this phenotype, suggesting that TBK1 may play an important role in radiation-induced EMT in normal alveolar epithelial cells.

### TBK1-dependent AKT phosphorylation is critical for radiation-induced EMT

**Fig. 3:**
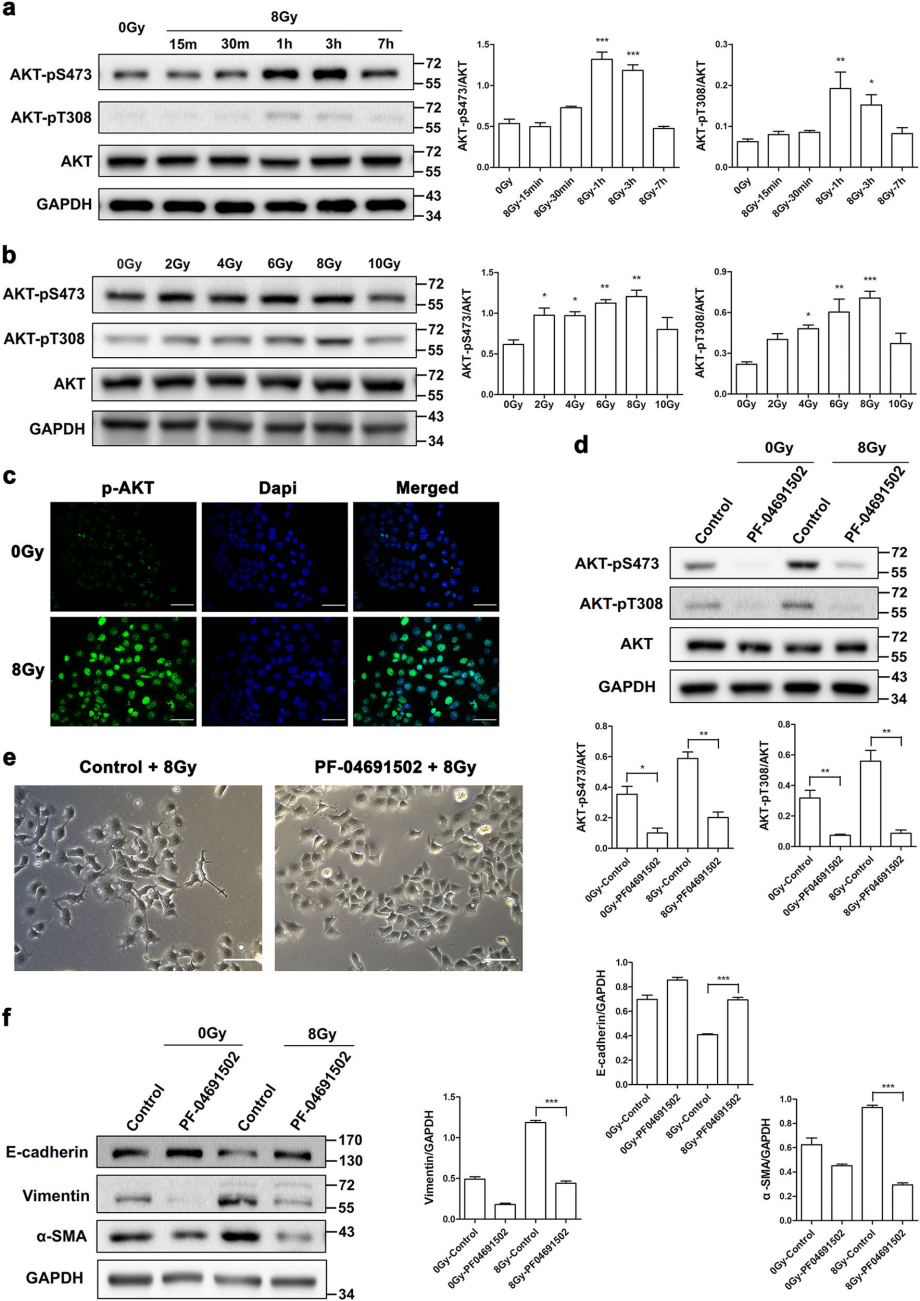
Inhibition of AKT attenuates radiation-induced changes in cell morphology and the expression of EMT markers. **a**, **b** Representative western blots and densitometric quantification of phosphorylated AKT on both S473 and T308 at different times and with different radiation doses. **c** Immunofluorescence staining for p-AKT (green) and DAPI staining (blue) in nonirradiated control and irradiated cells at 1 h postirradiation. Scale bar represents 50 μm. **d**–**f** RLE-6TN cells were incubated with/without the selective AKT inhibitor PF-04691502 for 2 h before irradiation with 8 Gy. **d**, **f** Western blot analysis of p-AKT, E-cadherin, vimentin, and α-SMA expression at 1 or 48 h postirradiation. **e** Phase contrast microscopy images of cells at 48 h after treatment with 8 Gy irradiation. Scale bar represents 100 μm. All the above western blot analyses use GAPDH or AKT as the loading control. The data are presented as the mean ± SEM (*n* = 3). *P < 0.05, **P < 0.01, and ***P < 0.001 vs. nonirradiated control

It is known that the AKT signaling pathway plays an important role in mediating EMT and is directly activated by TBK1 in cancer cells. Therefore, we aimed to test whether radiation could activate AKT and to determine its role in radiation-induced EMT. As shown in Fig. 3a, b, we found that AKT was significantly phosphorylated on both S473 and T308 at 1–3 h postirradiation and observed a dose-dependent increase in AKT activation with 2–8 Gy γ-ray treatment at 1 h postirradiation. Phosphorylation of AKT was further confirmed by immunofluorescence analysis (Fig. 3c). Then, to examine whether AKT participates in radiation-induced EMT in RLE-6TN cells, we preincubate cells with the selective AKT inhibitor PF-04691502 for 2 h before irradiation. As shown in Fig. 3d, PF-04691502 remarkably inhibited phosphorylation of AKT at 1 h postirradiation with 8 Gy irradiation. The cell morphology again showed a cuboidal appearance at 48 h postirradiation when treated with the AKT inhibitor (Fig. 3e). The protein levels of E-cadherin, vimentin, and α-SMA were effectively modulated by PF-04691502 at 48 h postirradiation (Fig. 3f), which indicated that blocking of AKT inhibited radiation-induced EMT in RLE-6TN cells.

**Fig. 4:**
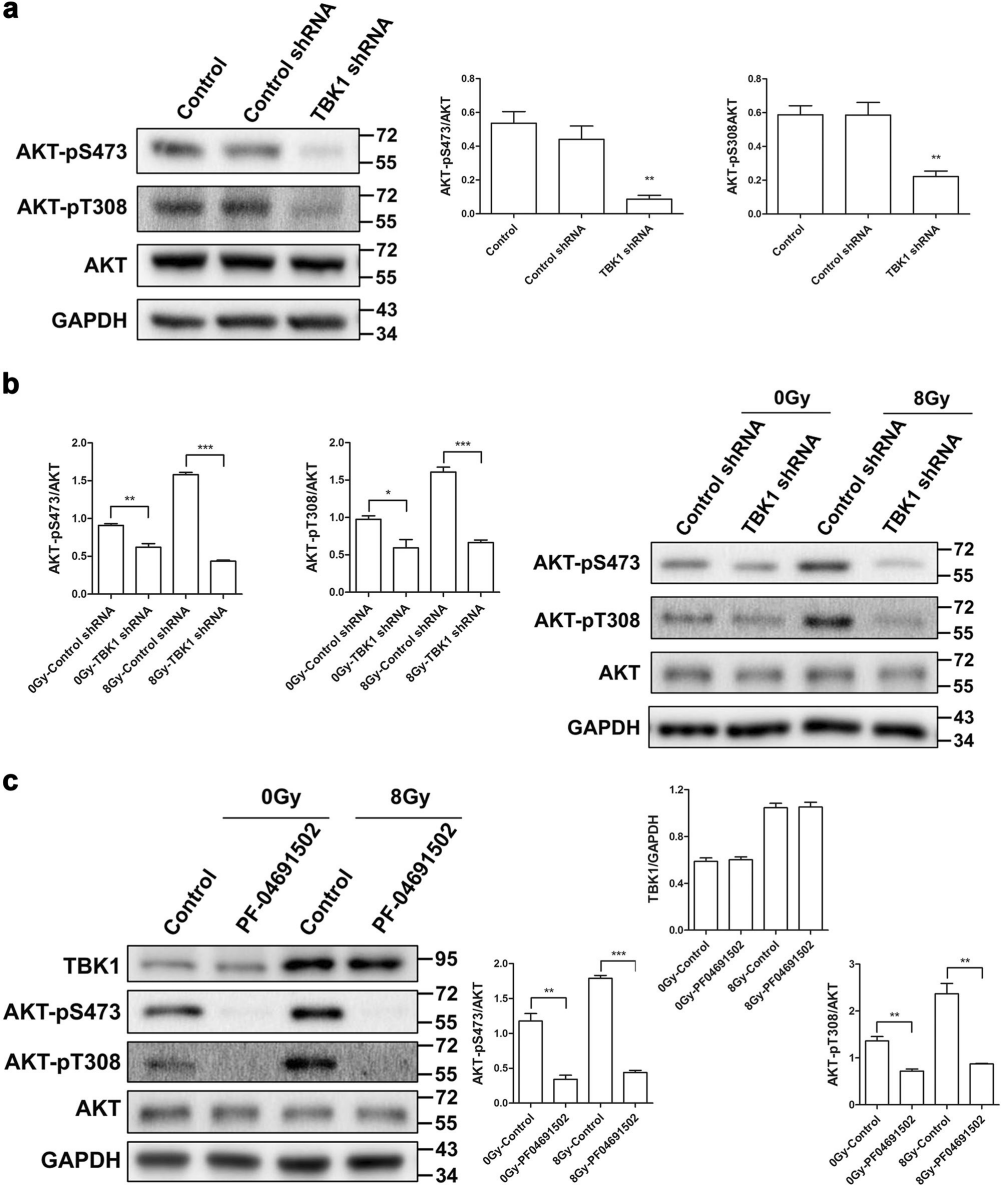
The involvement of TBK1 in radiation-induced EMT occurs through the AKT signaling pathway. **a**, **b** The expression levels of AKT-pS473 and AKT-pT308 in TBK1 knockdown RLE-6TN cells treated with or without 8 Gy irradiation were detected via western blot analysis. GAPDH and AKT were used as loading controls. **c** RLE-6TN cells were incubated with/without PF-04691502 for 2 h before 8 Gy irradiation. Cell lysates were collected, and the protein levels of AKT-pS473, AKT-pT308, and TBK1 at 1 h postirradiation were measured via western blot. GAPDH and AKT were used as loading controls. The data are presented as the mean ± SEM (*n* = 3). *P < 0.05, **P < 0.01, and ***P < 0.001 vs. control group

Then, we investigated the possibility of a relationship between TBK1 and AKT. As shown in Fig. 4a, TBK1 shRNA blocked AKT phosphorylation on both S473 and T308, but the level of total AKT was not altered. Furthermore, silencing of TBK1 decreased the activation of AKT induced by 8 Gy irradiation at 1 h postirradiation (Fig. 4b). In contrast, the AKT inhibitor PF-04691502 had little effect on the protein level of TBK1, even after irradiation (Fig. 4c). These data indicate that AKT might be downstream of TBK1, suggesting that TBK1 may promote radiation-induced EMT through the AKT signaling pathway.

### TBK1 is required for radiation-induced ERK activation and EMT

**Fig. 5:**
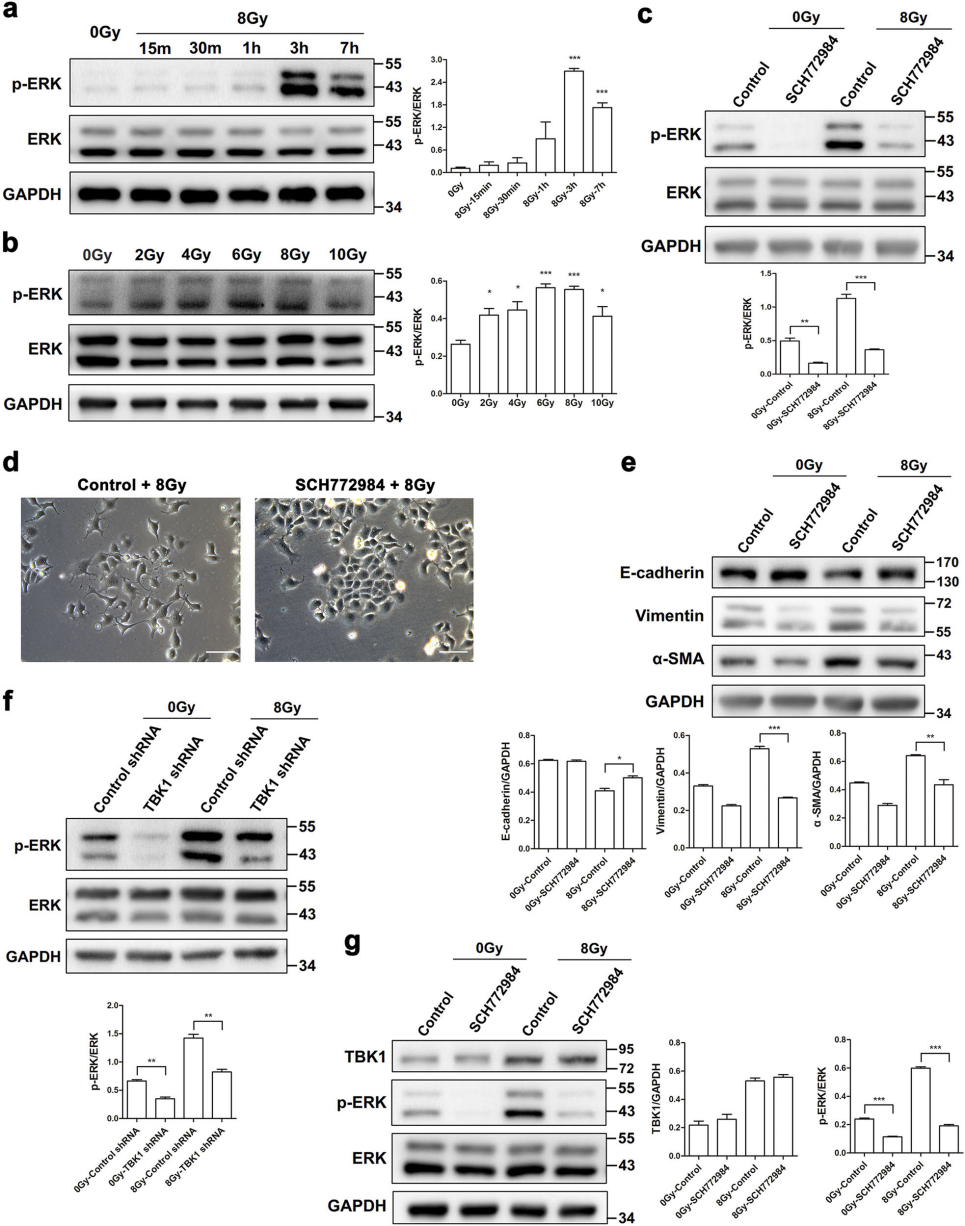
The ERK signaling pathway accounts for the regulatory effects of TBK1 in radiation-induced EMT **a**, **b** Representative western blots and densitometric quantification of phosphorylated ERK at different times and with different radiation doses. **c**–**e** RLE-6TN cells were incubated with/without the selective ERK inhibitor SCH772984 for 2 h before irradiation with 8 Gy. **c**, **e** Western blot analysis of p-ERK, E-cadherin, vimentin, and α-SMA at 1 or 48 h postirradiation. **d** Phase contrast microscopy images of cells at 48 h after treatment with 8 Gy irradiation. Scale bar represents 100 μm. **f** The expression level of p-ERK in TBK1 knockdown RLE-6TN cells treated with or without 8 Gy irradiation was detected by western blot analysis. **g** RLE-6TN cells were incubated with/without SCH772984 for 2 h before irradiation with 8 Gy. Cell lysates were collected, and the protein levels of p-ERK and TBK1 at 3 h postirradiation were measured by western blot. GAPDH and ERK were used as loading controls. The data are presented as the mean ± SEM (*n* = 3). *P < 0.05, **P < 0.01, and ***P < 0.001 vs. nonirradiated control

It was also revealed that ERK signaling was involved in EMT. Therefore, we infer that there may be some connection between TBK1 and ERK in mediating radiation-induced EMT. We observed that ERK was activated by irradiation in a time-dependent and dose-dependent manner. As revealed in Fig. 5a, ERK was significantly phosphorylated at 3 and 7 h after treatment with 8 Gy irradiation. Consistently, ERK phosphorylation was also observed in a dose-dependent manner ranging from 2 to 10 Gy, particularly with 6 and 8 Gy irradiation (Fig. 5b). The ERK selective inhibitor SCH772984 markedly blocked radiation-induced phosphorylation of ERK (Fig. 5c), and thus reversed the EMT phenotype in irradiated cells (Fig. 5d).

To investigate whether radiation-induced phosphorylation of ERK requires TBK1, we examined the phosphorylation of ERK in TBK1 knockdown cells. TBK1 knockdown restrained the basal level of phosphorylated ERK (Fig. 5e) and sharply inhibited radiation-induced phosphorylation of ERK at 3 h postirradiation (Fig. 5f). However, blocking ERK did not affect the protein level of TBK1 (Fig. 5g). Thus, ERK might be downstream of TBK1. These data indicate that the radiation-induced EMT in RLE-6TN cells is mediated, at least in part, via the TBK1–ERK signaling pathway.

### AKT-mediated ERK signaling downstream of TBK1 is critical for radiation-induced EMT

**Fig. 6:**
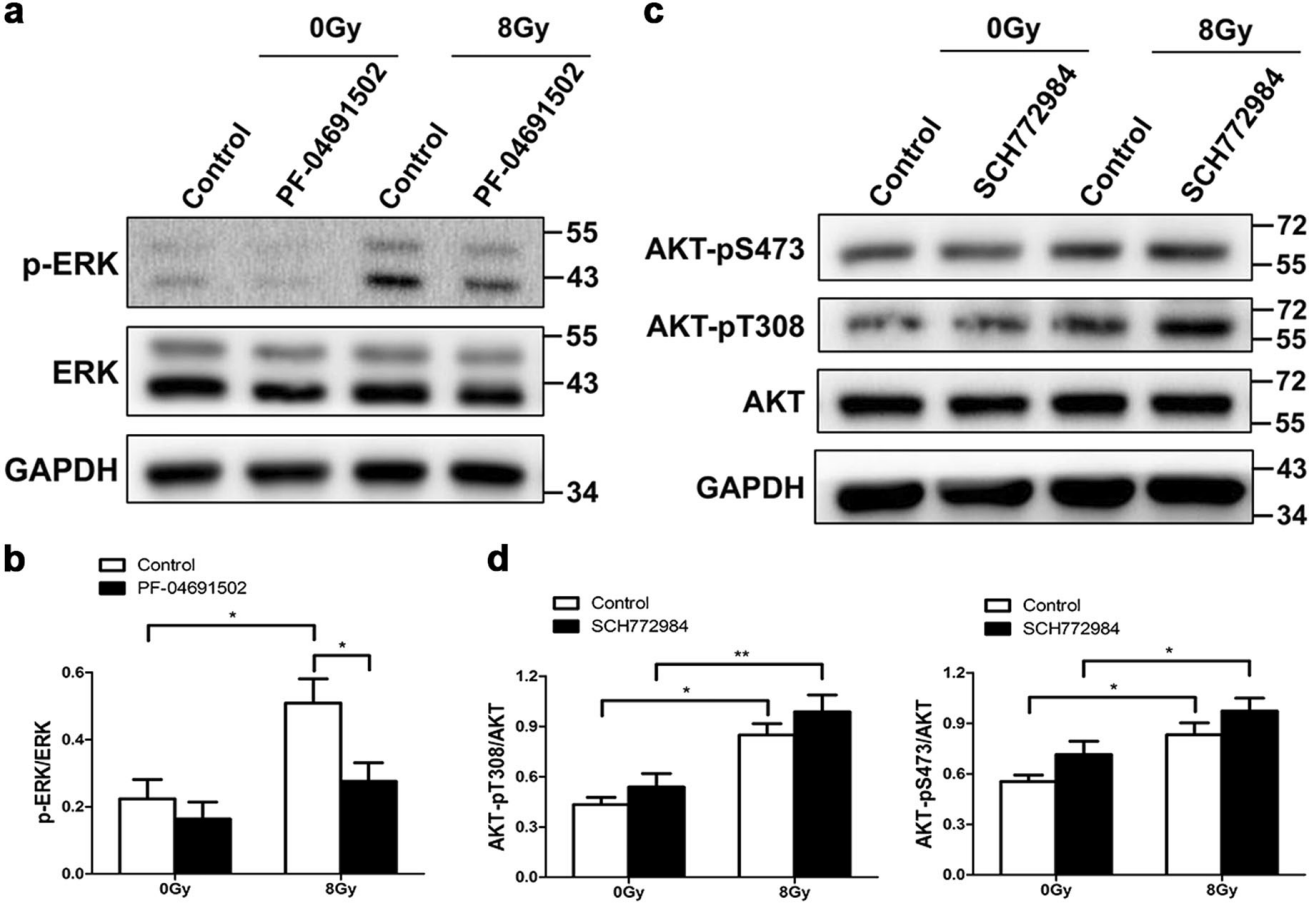
AKT-mediated ERK signaling is critical for radiation-induced EMT downstream of TBK1. RLE-6TN cells were incubated with/without inhibitor for 2 h before irradiation with 8 Gy. **a**, **b** Representative western blot and densitometric quantification of p-ERK at 3 h postirradiation. GAPDH and ERK were used as loading controls. **c**, **d** Representative western blot and densitometric quantification of AKT-pS473 and AKT-pT308 at 1 h postirradiation. GAPDH and AKT were used as loading controls. All data are presented as the mean ± SEM (*n* = 4). One-way ANOVA with Newman–Keuls post hoc analysis was used. *P < 0.05 and **P < 0.01

We found that AKT and ERK signaling downstream of TBK1 may be necessary for radiation-induced EMT in RLE-6TN cells. Further, we investigated the relationship between AKT and ERK signaling in this biological process. Western blot analyses showed that phosphorylation of ERK was significantly decreased in irradiated RLE-6TN cells pretreated with the AKT inhibitor PF-04691502, whereas the levels of total ERK remained unchanged (Fig. 6a, b). This result indicated that AKT inhibition could override the radiation-induced activation of ERK. Then, we used the ERK inhibitor SCH772984 to confirm whether blocking ERK could attenuate radiation-induced AKT phosphorylation. However, there was no difference in the phosphorylated AKT protein level between nonirradiated control cells and those pretreated with the inhibitor (Fig. 6c, d). Nevertheless, SCH772984 did not obviously affect radiation-induced phosphorylation of AKT (Fig. 6c, d). Although there was some complex crosstalk between AKT and ERK signaling, these data suggest that AKT might act upstream of ERK in radiation-induced EMT in RLE-6TN cells.

### The TBK1 inhibitor Amlexanox alleviated radiation-induced pulmonary injury and fibrosis

**Fig. 7:**
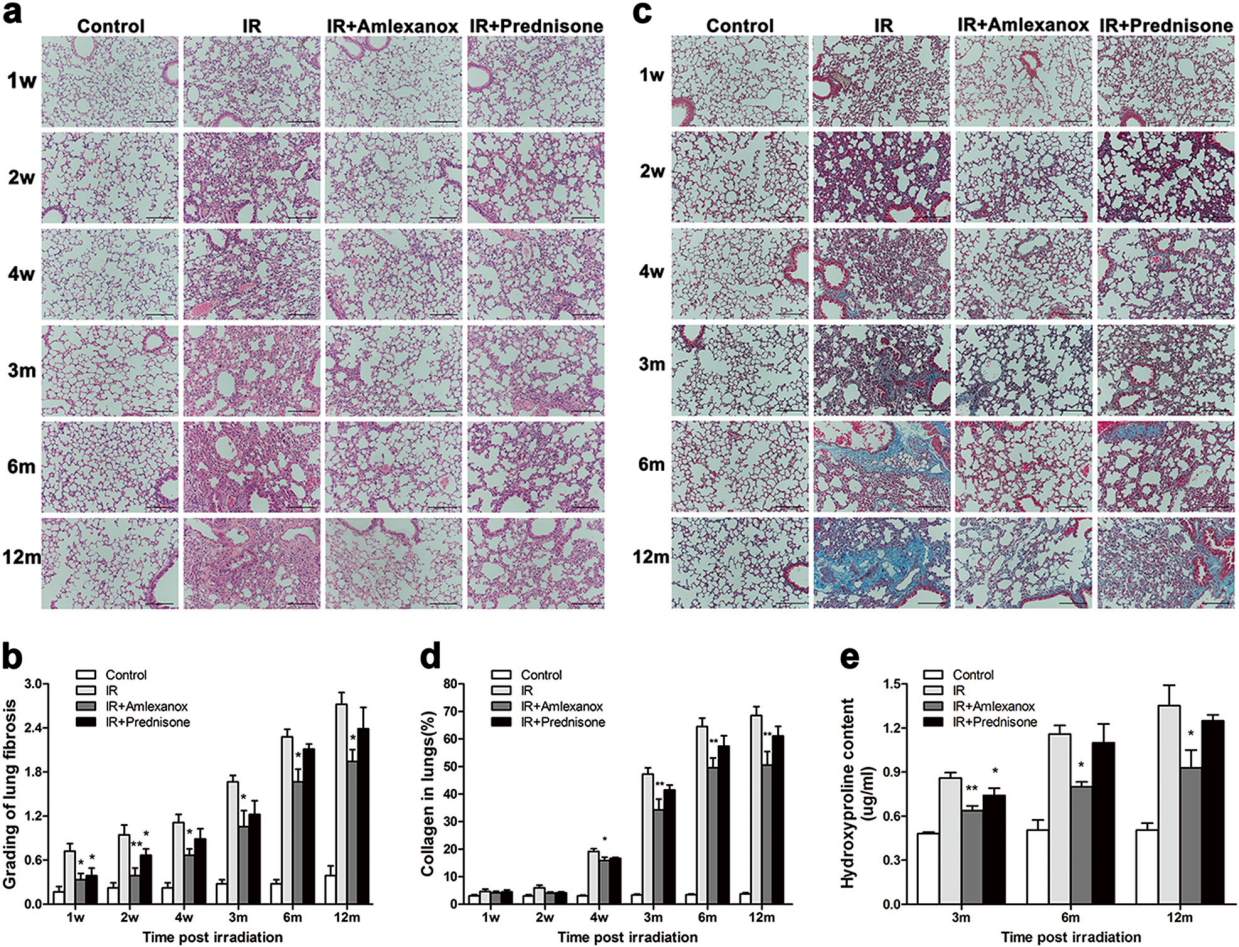
The TBK1 inhibitor Amlexanox alleviated radiation-induced pulmonary injury and fibrosis. The lung area of mice was exposed to local irradiation at a dose of 15 Gy, and HE (**a**) and Masson staining (**c**) were conducted at 1 to 12 months postirradiation. Lung fibrosis (**b**) and collagen (**d**) deposition were quantified in lung sections from different groups. At different time points, hydroxyproline content was measured via ELISA in different groups (**e**). *P < 0.05 and **P < 0.01

EMT is a critical process in radiation-induced lung fibrosis, which is one of the most important complications of lung cancer radiotherapy. Because TBK1 was found to play a critical role in EMT, we investigated the potential therapeutic role of TBK1 in RILI. We used the TBK1 inhibitor Amlexanox and found that Amlexanox obviously attenuated lung injury (Fig. 7a) and collagen deposition (Fig. 7c) at 1 week, 2 weeks, 4 weeks, 3 months, 6 months, and 12 months after irradiation. Quantitative data showed that Amlexanox significantly reduced lung fibrosis (Fig. 7b) and collagen deposition in lung tissues (Fig. 7d). Then, we measured the hydroxyproline content in lung tissues from different groups and found that Amlexanox significantly reduced the level of hydroxyproline after IR (Fig. 7e). In terms of lung fibrosis, the protective effects of Amlexanox were better than that in the prednisone group, the therapeutic medicine presently used for radiation-induced pulmonary injury (RIPI).

### Amlexanox inhibited EMT in lung tissues after irradiation

**Fig. 8:**
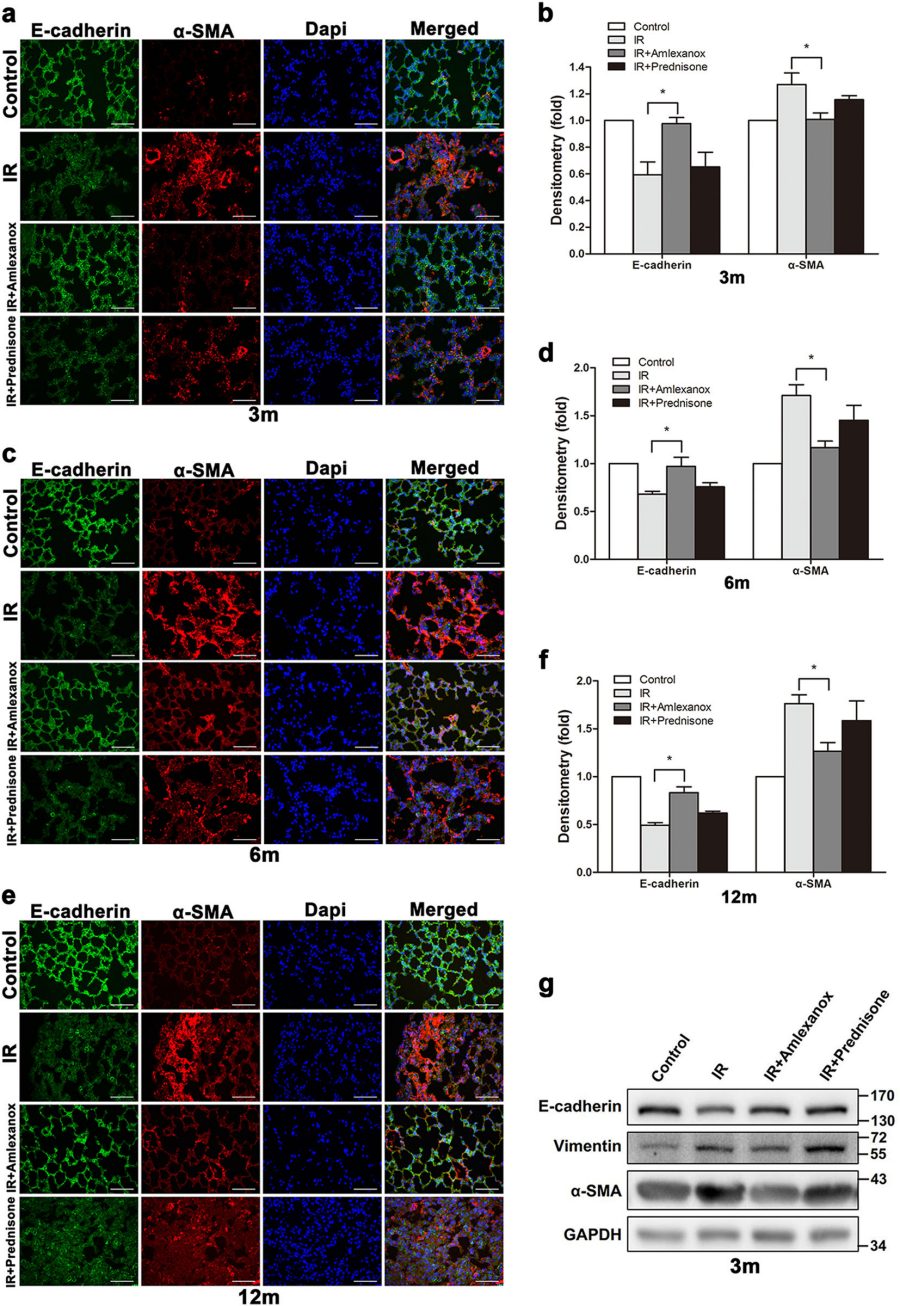

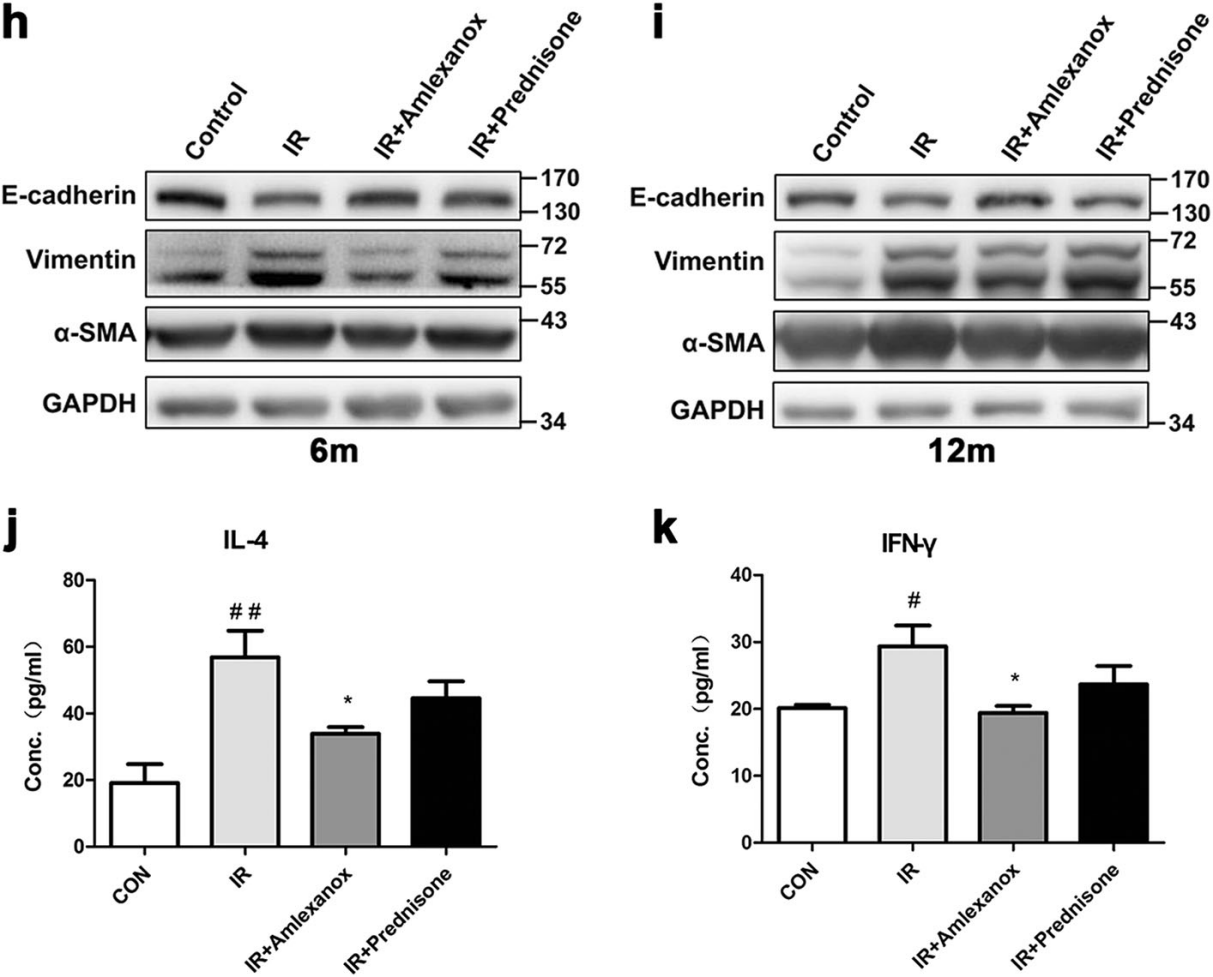
Amlexanox inhibited EMT in lung tissues after irradiation. At 3, 6, and 12 months after irradiation, lung tissues were isolated, and stained for EMT-related markers, including E-cadherin and α-SMA (**a**, **c**, **e**); fluorescence density was quantified in each group using software (**b**, **d**, **f**). *P < 0.05 and **P < 0.01. Expression levels of the EMT markers E-cadherin, Vimentin, and α-SMA were confirmed by western blot assay at 3 (**g**), 6 (**h**), and 12 (**i**) months after irradiation. Cytokines, such as IL-4 (**j**) and IFN-γ (**k**), were measured in bronchoalveolar lavage fluid (BALF) via ELISA 3 months after irradiation. ^##^*P* < 0.01 and ^#^*P* < 0.05 vs. control group. *P < 0.05 vs. IR group

To examine the role of TBK1 in radiation-induced EMT in vivo, we assessed the expression of EMT-related markers in different groups after irradiation. Our data showed that local lung irradiation resulted in obvious EMT changes, such as downregulation of E-cadherin and upregulation of Vimentin and α-SMA. We found that Amlexanox significantly inhibited the decrease in E-cadherin and the upregulation of α-SMA at 3, 6, and 12 m after irradiation (Fig. 8a–f). The expression levels of these proteins were confirmed by western blot analysis (Fig. 8g–i). Inflammatory cytokines are important factors in radiation-induced lung fibrosis, and we checked the level of IL-4 and IFN-γ in BALF. Our data showed that Amlexanox significantly inhibited the radiation-induced increase in the levels of these cytokines (Fig. 8j, k).

### Amlexanox protected cells and mice against lethal radiation damage

**Fig. 9:**
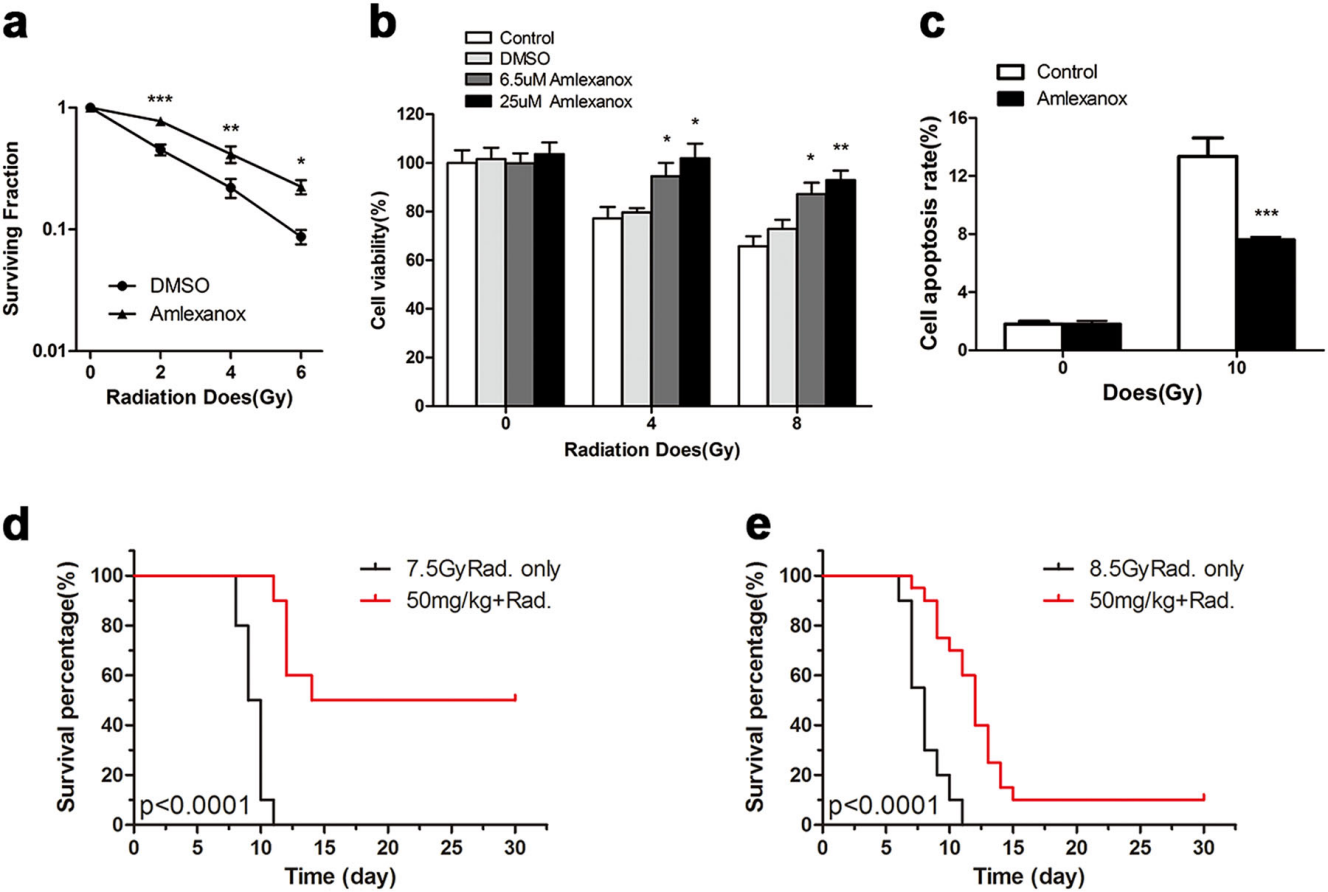
The TBK1 inhibitor Amlexanox protected cells and mice against lethal radiation damage. After 2, 4, 6, or 8 Gy single-dose irradiation, cell survival was determined in the radiation- and Amlexanox-treated groups via colony formation (**a**) and CCK-8 (**b**) assays. Cell apoptosis was measured by flow cytometry 24 h after 10 Gy irradiation (**c**). For in vivo study, BALB/c mice with/without Amlexanox treatment were exposed to 7 or 8.5 Gy irradiation, and animal survival was monitored for up to 30 days after irradiation (**d**, **e**). *P < 0.05, **P < 0.01, and ***P < 0.001 vs. nonirradiated control

To investigate whether Amlexanox exerts protective effects in normal cells and mice, we assessed cell survival in response to different doses of radiation. It was found that Amlexanox significantly increased cell survival after 0, 2, 4, and 8 Gy irradiation (Fig. 9a), which was also confirmed by CCK-8 assays (Fig. 9b). Next, we observed that radiation-induced cell apoptosis in RLE-6TN cells was significantly inhibited by Amlexanox treatment (Fig. 9c). Then, we treated mice with 50 mg/kg Amlexanox before irradiation and found that animal survival was significantly increased in both the 7.5 and 8.5 Gy-irradiated groups (Fig. 9d, e).

## Discussion

As a serious complication of thoracic radiotherapy, RIPF is characterized by excessive fibroblast proliferation and massive deposition of extracellular matrix and causes severe physiologic abnormalities and chronic respiratory failure in patients^28,29^. Although fibroblast/myofibroblast activation has been recognized as an important contributor, the exact mechanisms underlying lung fibrosis remain elusive. Recently, experiments using genetic cell fate tracking of lung epithelial cells were performed to investigate this issue in a mouse model of lung fibrosis. These studies found that approximately 30–50% of murine lung fibroblasts in lung fibrosis were derived from epithelial cells that had undergone EMT as identified by genetic tagging^30–32^. Emerging evidence suggests that injured epithelial cells suffering EMT are important sources of myofibroblasts in fibrosis^33^. In this study, we confirmed that radiation-induced EMT in normal alveolar epithelial cells. Our results showed time-dependent and dose-dependent increases in the expression of TBK1, p-AKT, and p-ERK after radiation, and blockage of these proteins reversed radiation-induced EMT, suggesting that they play a crucial role in this process. To the best of our knowledge, this is the first report showing that TBK1 is upstream of ERK and the TBK1–AKT–ERK signaling pathway promotes radiation-induced EMT in normal alveolar epithelial cells.

Recently, cumulative studies have demonstrated that the occurrence of radiation-induced EMT in malignant cancer cells is closely related to their invasive potential. In contrast, only a few groups have conducted studies on normal alveolar epithelial cells undergoing radiation-induced EMT^13,16^. It is well known that EMT has been classified into three subtypes according to its functional consequences and biomarkers: Type 1 EMT is EMT during implantation, embryogenesis, and organ development; Type 2 EMT is concerned with tissue regeneration and organ fibrosis; Type 3 EMT is associated with cancer progression and metastasis^34,35^. Radiation is a double-edged sword that not only kills tumor cells but can also damage normal tissues. Here, we sought to study the underlying mechanism of radiation-induced Type 2 EMT in normal alveolar epithelial cells. Nagarajan et al.^13^ first reported radiation-induced EMT in normal rat type II alveolar RLE-6TN cells. Then, Xiong et al.^16^ examined radiation-induced EMT in normal mouse alveolar epithelial MLE 12 cells. In the present study, we demonstrated that radiation-induced EMT is closely related to different radiation doses and time points. In addition to traditional EMT markers, we also quantified the number of cells with obvious morphology changes according to three criteria: swelling, elongation, and extension of pseudopodia from cells vs. a cuboidal appearance. From 0 to 10 Gy, the number of cells undergoing EMT was highest in the 8 Gy group, while with 10 Gy irradiation, cell death was induced in more cells. We provided a comprehensive analysis of radiation-induced EMT in normal type II alveolar cells.

TBK1, a noncanonical member of the inhibitor B (IκB) kinase-related kinase (IKK) family, participates in inflammatory pathways related to activation of NF-κB, hosts cytokine production, and plays a crucial role in Ras-induced oncogenesis and in autophagy^36–39^. Additionally, our previous study confirmed that TBK1 promotes radiation-induced Type 3 EMT in lung cancer A549 cells. In the present study, we found that the TBK1 level was increased in a time-dependent and dose-dependent manner after irradiation in normal alveolar RLE-6TN cells. Western blot analysis showed that the loss of TBK1 downregulated the epithelial marker E-cadherin and increased the mesenchymal markers Vimentin and α-SMA. Our data suggested that TBK1 induced radiation-induced Type 2 EMT in normal alveolar epithelial cells.

Interestingly, our previous findings indicated that TBK1 promotes radiation-induced EMT through inactivation of GSK3β and activation of ZEB1 (ref. ^14^). Previous studies demonstrated that AKT is directly phosphorylated by TBK1 and mediates its prosurvival role^26,27^. The ERK/MAPK and PI3K/Akt pathways mediate Ras mutant-induced Type 3 EMT, which is reversed by either wild-type Ras or MAPK kinase 1 (MEK1) inhibitor^40^. Moreover, activated ERK causes phosphorylation of GSK3β, resulting in unbound Snail migrating to the nucleus and eventually leading to EMT^13^. Therefore, we wondered whether TBK1 could regulate radiation-induced Type 2 EMT via AKT and ERK signaling in normal alveolar epithelial cells. We investigated the response of the AKT and ERK signaling pathway to radiation in RLE-6TN cells. Phosphorylation of both AKT and ERK was triggered in a time-dependent and dose-dependent manner in response to irradiation. Moreover, blocking of AKT or ERK abolished radiation-induced EMT, indicating that AKT and ERK signaling are both involved in radiation-induced EMT. The relationship between TBK1 and AKT has been studied for several years, but few report have investigated the crosstalk between TBK1 and ERK. There is no direct evidence showing a role of TBK1 in activation of either AKT or ERK. In TBK1 knockdown cells, we found that radiation-induced phosphorylation of AKT and ERK was inhibited. Conversely, selective inhibitors of AKT or ERK dramatically blocked phosphorylation of AKT and ERK but did not affect the level of TBK1, indicating that AKT and ERK signaling might be downstream of TBK1. As a result, our data suggest that TBK1 signaling may be necessary for regulation of radiation-induced EMT via AKT and ERK signaling in normal alveolar epithelial cells. Then, we focused on the relationship between AKT and ERK. Previously, it was reported that these two signaling pathways exerted opposing effects and that the PI3K–AKT pathway inhibits the Raf–MEK–ERK pathway in muscle cell hypertrophy and breast cancer cell proliferation^41,42^. In contrast, we observed that inhibition of AKT signaling decreased the radiation-induced increase in p-ERK, while blockage of ERK signaling did not obviously change the expression of p-AKT. These data suggest that AKT might exert an upstream regulator of ERK in radiation-induced EMT, but the exact mechanism still needs to be clarified.

According to previous studies and our present findings, radiation-induced EMT in RLE-6TN cells is mediated, in part, through the TBK1–ERK–AKT signaling pathway. However, our study has some limitations. As a crucial signaling factor in EMT, transforming growth factor-β (TGF-β) is heavily implicated in radiation-induced fibrosis^43^. However, our previous findings suggested a lack of crosstalk between TBK1 signaling and the TGF-β/Smad pathway in radiation-induced EMT in lung cancer A549 cells, and a previous study has shown that ERK is less likely to be involved in TGF-β-induced human alveolar EMT^44^. In addition, whether ZEB1, Snail, and other major transcription factors, such as TWIST1, participate in this signaling pathway still needs to be validated. Cell culture models are beneficial for exploring the mechanism of EMT, lung fibrosis, and the associated treatment strategies.

The critical role of TBK1 in radiation-induced EMT provided novel therapeutic potential for radiation pulmonary fibrosis. In the present study, by using a RIPI model established by our group, we investigated the possibility of blocking TBK1 to prevent RIPI. Our data confirmed that a TBK1 inhibitor alleviated lung injury as well as fibrosis and inhibited the EMT process. EMT is a major process in fibrosis in many organs, and we also found that TBK1 inhibited EMT and reduced collagen deposition in lung tissues. As Th1 and Th2 balance play critical roles in lung fibrosis, we further examined the impact of TBK1 depletion on the Th1/Th2 cytokine balance in BALF after irradiation^45^. As is shown in our results, the levels of IFN-γ and IL-4 in BALF increased after radiation, while TBK1 inhibitor restricted the upregulation of these cytokines. Finally, we found that Amlexanox protects normal cells and mice against irradiation. These data provide novel insights into potential therapy for RIPF in normal tissues, which will be beneficial for delivery of radiotherapy in cancer patients.

In conclusion, we have shown that inhibition of TBK1, AKT, and ERK signaling suppressed radiation-induced EMT in RLE-6TN cells. In particular, TBK1, as a significant upstream signaling molecule, regulates radiation-induced EMT through AKT-ERK signaling. Our in vivo data showed that a TBK1 inhibitor alleviates RIPI and protects mice against IR, providing a novel strategy for preventing RIPI during cancer radiotherap.
